# Systematic Review of Executive Function Stimulation Methods in the ADHD Population

**DOI:** 10.3390/jcm13144208

**Published:** 2024-07-19

**Authors:** Carlos Ramos-Galarza, Deyaneira Brito, Brayan Rodríguez, Brenda Guerrero, Jorge Cruz-Cárdenas, Mónica Bolaños-Pasquel

**Affiliations:** 1 Factultad de Psicología, Pontificia Universidad Católica del Ecuador, Quito 170525, Ecuador; dabritom@puce.edu.ec (D.B.); brodriguez776@puce.edu.ec (B.R.); bmguerrero95@gmail.com (B.G.); 2 Centro de Investigación Estec, Facultad de Administración y Negocios, Universidad Indoamérica, Quito 170301, Ecuador; jorgecruz@uti.edu.ec; 3 Centro de Investigación MIST, Universidad Indoamérica, Quito 170301, Ecuador; monika_martina@hotmail.com

**Keywords:** attention deficit hyperactivity disorder, executive functions, treatment

## Abstract

**Background/Objectives:** Attention deficit hyperactivity disorder (ADHD) is a neurodevelopmental disorder characterized by elevated motor activity, impulsivity, and attention deficit. Approximately 5% of the population suffers from this disorder. Among the key explanations of ADHD, executive functions play an important role in understanding the symptomatology present in this disorder and in determining the main treatment strategies for affected patients. We present a systematic review that seeks to identify the treatment methods developed to support executive functions in individuals with ADHD. **Methods:** Articles were analyzed in the SCOPUS, PUBMED, and Science Direct databases. Initially, 739 articles were found. After applying inclusion and exclusion criteria, 30 articles remained and were included in the data extraction process. **Results:** Among the primary treatments identified, 14 studies propose psychological training for executive functions, 9 studies recommend medication, 5 studies suggest digital interventions, and 1 study advocates for sports as beneficial for executive functions. **Conclusions:** The data are discussed around the need to develop new proposals to enhance the executive functions of individuals with ADHD, thereby improving their performance in educational, personal, social, and family activities impacted by this disorder.

## 1. Introduction

ADHD is a neurodevelopmental disorder characterized by persistent patterns of inattention, hyperactivity, and impulsivity. These symptoms often manifest in childhood and can persist into adolescence and adulthood [1]. The global prevalence of ADHD ranges from 5.9% to 7.1% in children and 1.2% to 7.3% in adults, indicating its significant impact across different age groups [2,3].

However, prevalence rates of ADHD can vary based on ethnic differences. Contrary to previous beliefs, recent studies contradict the notion that the prevalence of ADHD is lower in certain ethnic groups. For instance, while it was once thought that Black children and adolescents had lower rates of ADHD compared to their White counterparts, research has shown that the prevalence rates between these groups do not significantly differ [4]. Moreover, Asian and Latino children and adolescents tend to present lower prevalence rates of ADHD than both their Black and White counterparts. These differences may be influenced by factors such as access to healthcare, socioeconomic status, and cultural attitudes toward mental health [5].

ADHD is a disorder of multifactorial origin, with the following most commonly described causal factors in the literature: genetic predispositions, gestational and perinatal factors, and environmental influences [2]. Changes in brain structure and function are observed in individuals with ADHD, affecting mechanisms such as neurogenesis and synaptogenesis, which, in turn, disrupt attention and impulse control, core functions regulated by the central nervous system [3]. The etiology of ADHD is diverse, with interactions between genetic and environmental factors contributing to the heterogeneous manifestation of the disorder among individuals and subsequently influencing their response to interventions [6].

This disagreement on the causes can contribute to a clash in treatment processes, which, in turn, impacts both academic and familial spheres. In the academic realm, for instance, children with ADHD may struggle to concentrate, follow instructions, and complete tasks, thereby affecting their academic performance and self-esteem [7]. Within the family, the lack of understanding about the nature of the disorder can generate stress and frustration, increasing tensions at home. This situation will affect the support networks available to a child with ADHD in their close environment since the specific needs of this population require collaborative efforts [8].

Individuals diagnosed with ADHD can encounter several challenges in their daily lives across various domains. In childhood and adolescence, ADHD can significantly impact academic achievement, social interactions, and overall well-being. Symptoms of ADHD, such as inattention and hyperactivity, often persist into adolescence, leading to difficulties in peer relationships, academic performance, and behavioral challenges. An estimated 72% of adolescents with ADHD present with sleep problems [9,10]. This affects their daytime functioning and exacerbates symptoms of inattention and hyperactivity. Moreover, untreated ADHD in adolescence increases susceptibility to risky behaviors, substance misuse, and mental health issues, including depression [11,12,13].

In adulthood, individuals with ADHD can face occupational challenges, educational impairments, financial difficulties, and family instability [14]. They are less likely to graduate from high school and college, have lower job stability, and demonstrate impaired job performance. ADHD also contributes to higher rates of substance abuse, mental health disorders, and financial stress, leading to increased reliance on public aid and decreased income [15,16].

Moreover, individuals with ADHD often experience comorbid conditions, such as anxiety disorders, significantly higher than the general population [17]. Anxiety symptom severity is associated with lower social skills and higher social problems in young people with ADHD, exacerbating existing social difficulties. Additionally, challenges with emotional regulation are common among individuals with ADHD, characterized by difficulties in managing and expressing emotions effectively. Studies indicate that adults with ADHD exhibit lower emotional regulation scores compared to controls, utilizing non-adaptive emotion regulation strategies such as self-blame and rumination. Emotional dysregulation is associated with greater socio-functional impairment, impacting relationships, stress management, and coping abilities. Addressing comorbid conditions and emotional regulation challenges is crucial for comprehensive support and intervention to mitigate the impact of ADHD [18,19].

One of the most widely accepted theories explaining the essential cause of ADHD symptomatology states that children, adolescents, or adults with this disorder present an immature executive function development [20]. Executive functions are a set of high-level mental abilities that help us plan, organize, and control behavior to achieve goals [21]. There is no specific number of executive functions and there are proposals that consider them as a single factor or multiple skills that interact to achieve behavioral regulation [22]. Different authors propose that there are different executive functions: inhibitory control, working memory, planning, verification, decision-making, emotional regulation, and cognitive flexibility [23,24,25].

In ADHD, the main executive function that is thought to be affected is inhibitory control. This impairment generates a domino effect, causing difficulties with working memory capacity, internal language regulating behavior, reconstitution of new behaviors, regulation of emotion, and arousal and motivation [26]. This alteration of the executive functions is what causes ADHD to present as a lack of regulation of behavior and cognition. This generates problems affecting individuals diagnosed with this disorder in different spheres, such as the educational, family, personal, social, work, and others [27,28,29].

The need to work on executive functions in ADHD is a crucial aspect of the management of this neurodevelopmental disorder. Over the years, various strategies have been explored to address these functions, which play a fundamental role in the regulation of behavior, attention, planning, and decision-making in individuals with ADHD. Among the most common interventions is the use of stimulant medications such as psychostimulants, which have been shown to improve attention and reduce hyperactive and impulsive symptoms in many patients. However, while these medications may be effective for some individuals, they are not a definitive solution as they do not directly address executive function deficits [30].

Another important approach is cognitive behavioral therapy (CBT), which focuses on identifying and changing dysfunctional patterns of thinking and behavior. CBT can be beneficial in improving skills such as self-regulation, problem-solving, and organization, which are directly related to executive functions. In addition, neuropsychological therapy, which focuses on rehabilitating specific areas of cognitive functioning, shows great potential for the treatment of ADHD. Through activities designed to improve working memory, cognitive flexibility, and inhibitory control, we seek to strengthen executive functions and improve overall functioning [31].

However, despite advances in these approaches, there is still no clear consensus on what is the best strategy for addressing impaired executive functions in ADHD. Research has shown mixed results leaving as a consequence many unanswered questions. Therefore, it is crucial to conduct a systematic review that integrates the most recent findings and evaluates the relative effectiveness of various interventions that address executive functions in people with ADHD.

This review could help inform clinical practice and guide the development of more effective and personalized future research that addresses the specific needs of each individual with ADHD. In addition, it would highlight the importance of further research in this field to improve the quality of life and functioning of individuals affected by this disorder.

## 2. Materials and Methods

**Figure 1:**
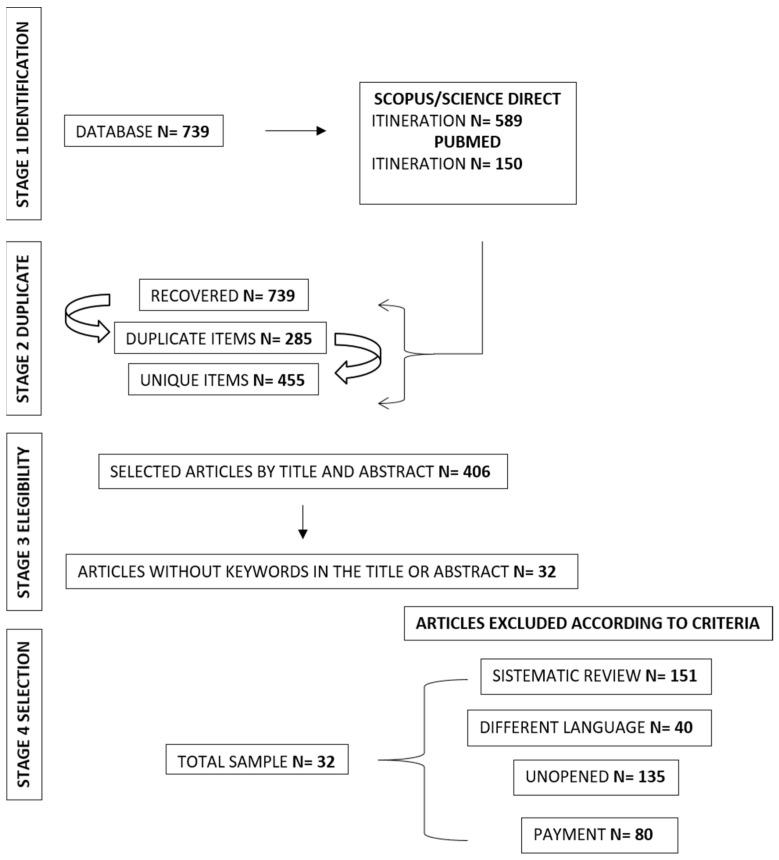
Flowchart of the systematic review conducted.

The scope of this research focuses on analyzing systematic reviews by applying the PRISMA method [32]. The first stage involved identifying the research question and proceeding with data collection. Initially, a total of N = 739 articles were obtained. In the second stage, duplicate articles (N = 285) were identified and excluded. In the third stage, the remaining articles were assessed according to the inclusion criteria (human participation, treatment to improve executive functions, people diagnosed with ADHD, and articles in English and Spanish) and exclusion criteria (languages other than Spanish and English, systematic reviews, books or theses, paid access, or articles that proposed interventions not aimed at improving executive functions in ADHD), resulting in the exclusion of N = 406 articles. Consequently, in the fourth stage, a selection of N = 32 articles was made and used in this research. In the fifth stage, the statistical analysis is presented. Finally, in the sixth stage, the summary collation and dissemination of the results are carried out through the application of the analytical framework (Figure 1).

To achieve our research objective, we used the following databases: SCOPUS, Science Direct, and PUBMED using the keywords: executive functions, ADHD, treatment, and intervention, with the respective Boolean terms (OR, AND). During the research process, three reviewers supervised: titles, abstracts, and full texts considering the inclusion and exclusion criteria, thus accepting, or rejecting the discrepancies found in the papers. Consequently, each independent article was combined into a single file, thus adopting the results of the three reviewers by extracting the files with different results. The extraction table included data such as authors, research design, year of publication, the sample size used, type of intervention, intervention time, country of application, tests applied to assess executive functions, and results, among others. This investigation has been registered in the PROSPERO platform under the number 557,233. This information is presented in Appendix A.

## 3. Results

The process to obtain the results involved a series of meticulous steps that allowed for a comprehensive analysis and understanding of the data. Firstly, dynamic tables were used to organize the data in a structured manner. These tables provide a dynamic view of the items for each variable, facilitating the identification of patterns and key relationships among the data. Subsequently, specific ranges were generated to perform detailed and segmented tabulations of the results. These ranges enabled us to group similar data and establish relevant categories for deeper analysis. Once the data were organized and analyzed, the results were visualized through charts. These charts were designed to visually represent the trends and relationships identified in the data, thus facilitating their interpretation.

Furthermore, key statistical measures, such as mean and standard deviation, were calculated for both subjects with ADHD for adults and children participating in the study. These measures provided us with a deeper understanding of the central tendency and dispersion of the data, enriching our analysis and conclusions. In summary, the process of obtaining results was a combination of descriptive and visual data analysis techniques that allowed us to explore and understand our research findings in depth.

### 3.1. Research Design

**Figure 2:**
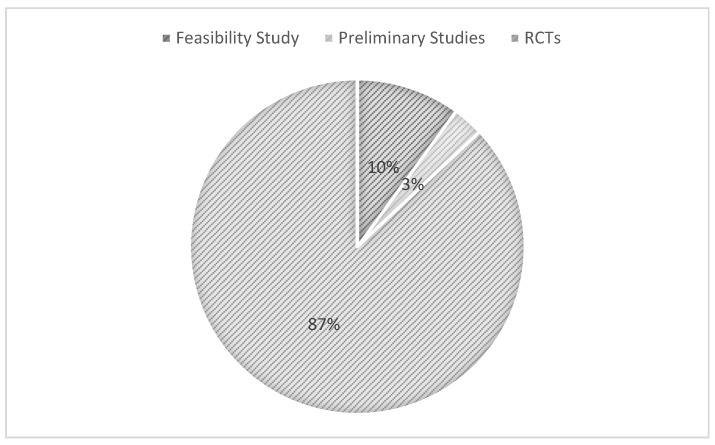
Research designs of the articles reviewed.

Feasibility studies, preliminary studies, and randomized controlled trials (RCTs) were the main research designs we encountered. RCTs stood out as the most prevalent. Check out Figure 2 to see their prominence.

### 3.2. Research Countries

**Figure 3:**
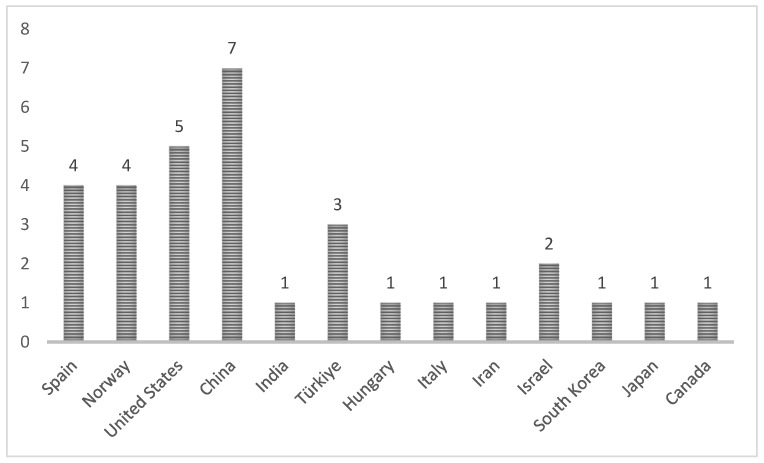
Research in countries.

China leads with 7 studies, followed by the United States with 5 and Norway with 4, among other countries. Discover this distribution of studies in Figure 3.

### 3.3. Participants Stage

**Figure 4:**
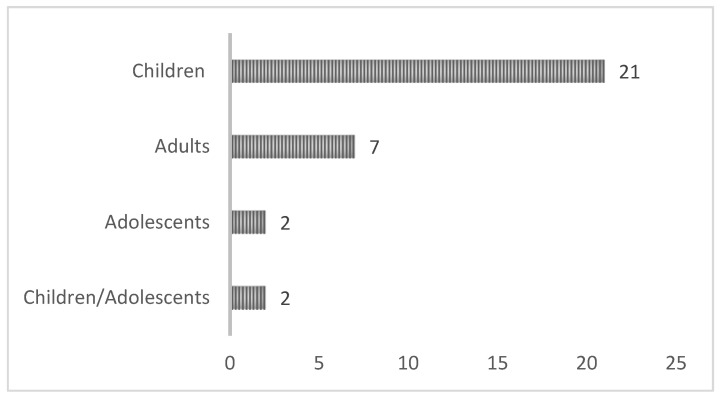
Subjects’ stage.

In terms of the life stage of individuals who underwent treatment, there were 21 studies involving children, followed by 7 studies in adults. Additionally, 2 studies encompassed adolescents and a combination of children and adolescents. These data are presented in Figure 4.

### 3.4. Treatment Implemented

**Figure 5:**
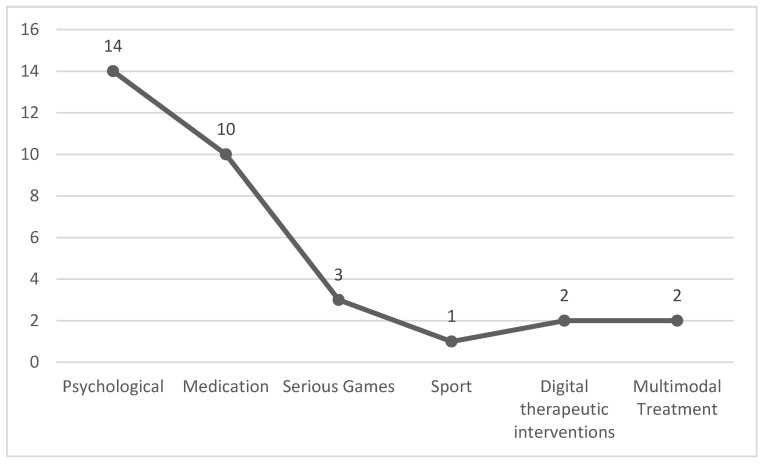
Treatment Type.

In the articles, various treatments were implemented to improve executive functions in individuals diagnosed with ADHD. The primary treatments included methylphenidate and mindfulness, followed by transcranial anodal direct current stimulation and viloxazine, among others. Psychological treatments were predominantly used, featured in 14 studies, followed by medication in 9 studies. Conversely, treatments such as polyamines and sports received the lowest number of studies. This distribution is visualized in Figure 5.

### 3.5. Test Administered in Previous Research

**Figure 6:**
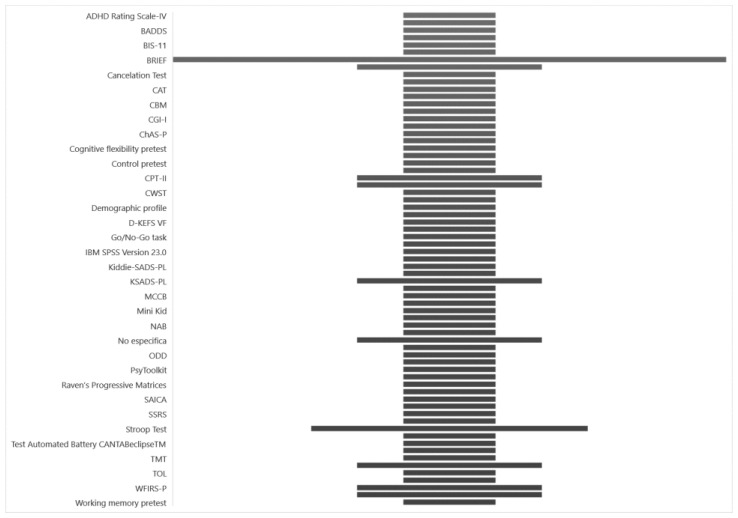
Test implemented.

In the articles, various tests were administered, with the BRIEF used in 6 studies and the Stroop test used in 3 studies. These tests were used to identify the presence of ADHD and assess executive functions. Explore Figure 6 to visualize the distribution of these tests across the studies.

### 3.6. Intervention Time

**Figure 7:**
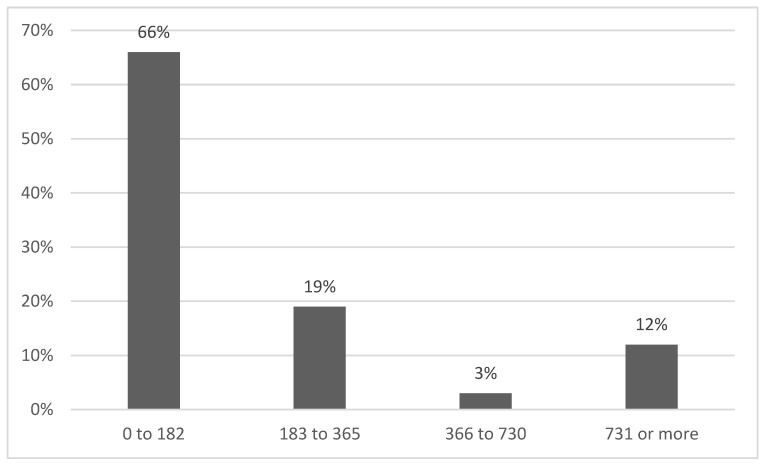
Implemented time in days.

There were four ranges used to identify the intervention duration of treatments in days. The range of 0–182 days included 21 studies, followed by 183–365 days and 731 days or more, each with 4 studies, and finally, 549–730 days with 1 study. Explore Figure 7 to visualize the distribution of treatment durations across the studies.

### 3.7. Improved Executive Functions

**Figure 8:**
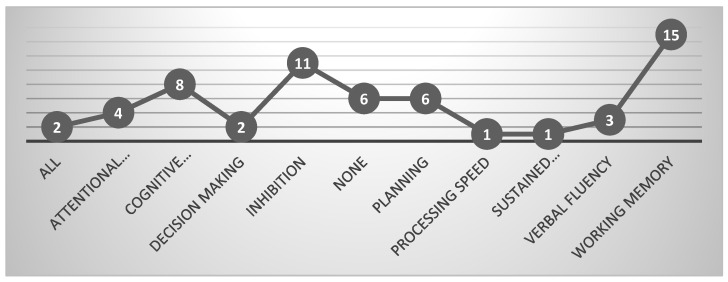
Improvement of executive functions.

In the analysis of the results, there was evidence of improvement in executive functions, although not uniformly across all aspects. Notably, working memory demonstrated the highest score in 15 studies, followed by inhibition in 12 studies, and cognitive flexibility in 5 studies. However, processing speed and sustained attention showed the lowest scores, each with only 1 study. To identify the efficacy of the treatment received with executive functions, a significant association was found between improving executive functioning and the type of psychological treatment (*x*^2^ = 11.82, *p* = 0.03). Explore these findings further in Figure 8.

## 4. Discussion

ADHD is a neurodevelopmental disorder that affects the normal development of children, adolescents, and adults. It is one of the most prevalent mental health problems. For this reason, it is vital to continue the dialogue on this matter and its treatment processes, which currently lack consensus on proposals or results [33,34].

Within the context of this article, the research based on a quantitative systematic review of treatment methods for executive functions in the ADHD population is presented. The importance of research on executive functions lies in the central role played by skills, such as inhibitory control, working memory, emotional regulation, cognitive flexibility, and planning in understanding the problems with the regulation of behavior and cognition that occur in this disorder [20,26].

The research began with 739 articles, and after analyzing the inclusion and exclusion criteria, 32 studies were included, allowing for the planned analyses to be carried out. The main results identified that randomized controlled trials were the most frequently performed research design. Regarding the temporality of studies, it was found that the last 5 years represent the highest scientific production in this line of research. Regarding the location of the research, it was found that the largest number of studies were conducted in Europe. As for the developmental level at which most studies were carried out, it was during childhood. The most frequently used treatment to support executive functions in ADHD was psychological interventions. The executive functions that benefit most from the identified treatments are working memory, inhibition, and cognitive flexibility. This study contributes to the understanding of ADHD by confirming the importance of executive functions when conducting interventions with individuals with this disorder. As previously mentioned, the roles of functions such as inhibitory control, working memory, and cognitive flexibility are crucial for individuals with ADHD to improve their behavioral regulation skills.

The findings of this systematic review underscore the breadth of research exploring interventions for ADHD, with particular emphasis on supporting executive functions, which tend to be affected by this neurodevelopmental disorder. Notably, both psychological interventions and pharmacotherapy have received substantial attention. For clinicians and healthcare providers navigating treatment options for individuals with ADHD, a thorough consideration of the available evidence must be conducted. Moreover, tailoring interventions to suit the developmental stage of the patient is essential to ensure the benefits seen in the patient’s quality of life. Furthermore, the combination of interventions, such as medication and psychological therapies, needs careful examination to ensure efficacy is grounded in empirical evidence.

Beyond treatment modalities, the cultural context emerges as a pivotal factor influencing intervention success. While existing research predominantly emanates from North America, Europe, and Asia, it is imperative to acknowledge and address the diversity of cultural backgrounds among individuals seeking treatment worldwide. Adapting interventions to local contexts is crucial for their applicability and efficacy [35,36,37]. This adaptation process necessitates not only clinical adjustments but also rigorous research to elucidate the specific cultural nuances that impact treatment engagement and effectiveness [38,39,40,41]. As such, bridging the gap between research and practice entails a comprehensive understanding of the multifaceted influences shaping the experiences of individuals with ADHD across diverse cultural landscapes.

Future research motivates us to develop our own intervention for executive functions in the ADHD population. Our interest lies in technological aspects that may be useful, especially for children and adolescents with the disorder. Finally, it is essential to continue studying executive functions, as these high-level mental abilities are not only affected in ADHD but also in other pathologies that affect human mental and behavioral activity and their pharmacological, non-pharmacological, and multimodal treatments [41,42,43,44,45,46,47,48,49,50,51].

The main limitation of this research lies in the lack of access to all the published articles, as several documents required payment to download. Another aspect is the language delimitation, as we only worked with studies published in English and Spanish; however, in future studies, we will review works published in other languages to cover the largest number of works on ADHD.

## Data Availability

The raw data supporting the conclusions of this article will be made available by the authors, without undue reservation.

## Appendix A

**Table A1:** Information Extraction Table.

Title	Country	Authors	Sample Size Used	Subjects with ADHD	Stage	Treatment Used	Type of Treatment	Test Implemented	Intervention Time/Days	Which Executive Functions are Improvement
Clinical and cognitive correlates of childhood attention-deficit/hyperactivity disorder in first-episode psychosis: a controlled study	Spain	Sánchez-Gistau et al. [52]	133	68	Children	Emotional stabilizers Antidepressants Antipsychotics	Medication	MCCB TMT-A NAB	1641	All
Dialectical behavioral therapy-based group treatment versus treatment as usual for adults with attention-deficit hyperactivity disorder: a multicenter randomized controlled trial	Norway	Halmøy et al. [53]	121	121	Adults	Mindfulness Dbt-bgt	Psychological	BRIEF DERS	98	All
Device-based movement behaviors, executive function, and academic skills among African-American children with ADHD and disruptive behavior disorders	United States	Santiago et al. [54]	42	23	Children	Af vigorosa	Psychological	BRIEF CBM AWMA-short version	7	None
Differential long-term medication impact on executive function and delay aversion in ADHD	Spain	Rubio and Hernández [55]	58	26	Children	Mph Atx	Psychological	K-BIT test Test automated battery cantabeclipsetm	273	Working memory Decision making Inhibition Planning Cognitive flexibility Verbal fluency
Effect of game-based high-intensity interval training program on the executive function of children with ADHD: protocol of a randomized controlled trial	China	Sun et al. [56]	42	42	Children	Gamehiit Games	Serious games	CWST CBTT Octamon fNIRS system TLT	60	Working memory Inhibition Planning Sustained attention Cognitive flexibility
Transcranial direct current stimulation as an effective treatment compared to video games on executive functions in children with attention deficit hyperactivity disorder	India	Makkar et al. [57]	61	61	Children	Tdcs along with video game Video game only	Serious games	TMT-A Raven progressive matrices	30	Working memory Cognitive flexibility Inhibition Verbal fluency
Effectiveness of online mindfulness-based intervention (imbi) on inattention, hyperactivity-impulsivity, and executive functioning in college emerging adults with attention-deficit/hyperactivity disorder: a study protocol	China	Pheh, et al. [58]	108	54	Adults	Mindfulness	Psychological	ASRS ADEXI	56	None
Effects of combing group executive functioning and online parent training on school-aged children with ADHD: a randomized controlled trial	China	Chu et al. [59]	145	145	Children	Gef-opt	Psychological	SNAP-IV BRIEF Go/no-go task WFIRS-P	30	Inhibition Planning Working memory
Effects of methylphenidate on executive functioning in children and adolescents with ADHD after long-term use: a randomized, placebo-controlled discontinuation study	Norway	Rosenau et al. [60]	94	94	Children and adolescents	Methylphenidate	Medication	Neuropsychological assessment	49	Working memory
Effects of agmatine, glutamate, arginine, and nitric oxide on executive functions in children with attention deficit hyperactivity disorder	Türkiye	Sarl et al. [61]	35	35	Children	Elisa	Medication	Stroop test TMT	365	None
Efficacy of cognitive behavioral therapy in medicated adults with attention-deficit/hyperactivity disorder in multiple dimensions: a randomized controlled trial	China	Pan et al. [62]	98	98	Adults	Methylphenidate	Medication	Cantab Regarding executive function	84	Inhibition
An empirical examination of executive functioning, ADHD associated behaviors, and functional impairments in adults with persistent ADHD, remittentADHD, and without ADHD	Spain	Roselió et al. [63]	61	61	Adults	Image	Psychological	CAARS BRIEF BRI MI WFIRS-P	1460	Inhibition Working memory Planning
Executive function and attention performance in children with ADHD: effects of medication and comparison with typically developing children	Hungary	Miklós et al. [64]	168	50	Children	Medication	Medication	Kitap testing Mini kid	730	Attentional control Inhibition
Executive function measured by brief in adolescents diagnosed and treated for ADHD: problem profiles and agreement between informants	Norway	Andersen et al. [65]	100	100	Adolescents	Tcc	Psychological	Kiddie-sads-pl Ksads-pl	1095	Working memory Planning
Executive function outcome of treatment with viloxazine extended-release capsules in children and adolescents with attention-deficit/hyperactivity disorder: a post-hoc analysis of four randomized clinical trials	United States	Faraone et al. [66]	1154	605	Children and adolescents	Viloxazine	Medication	Cgi-s Mmrm	56	All
Iamhero: preliminary findings of an experimental study to evaluate the statistical significance of an intervention for ADHD conducted through the use of serious games in virtual reality	Italy	Schena et al. [67]	60	60	Children	Iamhero	Serious games	Wisc-iv Bia Tol	42	Planning Attentional control Decision making
Feasibility, acceptability, and effectiveness of a new cognitive-behavioral intervention for college students with ADHD	United States	Solanto et al. [68]	19	19	Adolescents	Transcranial anodal direct current stimulation	Psychological	Home exercise	84	Working memory
										Cognitive flexibility
Improved executive function in adults diagnosed with attention-deficit/hyperactivity disorder as measured by the brown attention-deficit disorder scale following treatment with shp465 mixed amphetamine salts extended-release: post hoc analyses from 2 randomized, placebo-controlled studies	United States	Brown et al. [69]	673	673	Adults	Shp465 mas	Medication	Badds	42	Attentional control
Impacts of soccer on executive function in boys with ADHD	China	Chen [70]	968	968	Children	Soccer practice intervention	Sport	Control pretest Working memory pretest Cognitive flexibility pretest	42	Cognitive flexibility Inhibition
Influence of methylphenidate on long-term neuropsychological and everyday executive functioning after traumatic brain injury in children with secondary attention problems	United States	Leblond et al. [71]	26	26	Children	Methylphenidate	Medication	BRIEF CPT-II D-KEFS VF WISC-IV	168	Processing speed Attentional control
Mindfulness training for children with ADHD and their parents: a randomized control trial	Spain	Valero et al. [72]	30	30	Children	My mind program	Psychological	CONNERS WISC-IV NEPSY-II	168	Cognitive flexibility Working memory
Osmotic release oral system-methylphenidate hydrochloride (oros-mph) versus atomoxetine on executive function improvement and clinical effectiveness in ADHD: a randomized controlled trial	Türkiye	Torun et al. [73]	135	95	Children	Oros-mph	Medication	KSADS-PL CTRS Stroop test Cancelation test	126	None
Perceptual-motor skills reconstruction program improves executive functions in children with attention-deficit/hyperactivity disorder	Iran	Kouhbanani and Rothenberger [74]	50	50	Children	The perceptual-motor skills reconstruction program	Psychological	Demographic profile Clinical interview Conner’s parent rating scale-revised Delis-Kaplan executive function system	168	Cognitive flexibility Inhibition Verbal fluency Working memory
Parental occupation executive training (poet): an efficient innovative intervention for young children with attention deficit hyperactive disorder	Israel	Frisch et al. [75]	72	72	Children	Copm	Psychological	CPRS CTRS CHAS-P	84	Inhibition Working memory
The potential effectiveness of digital therapeutics specialized in executive functions as an adjunctive treatment for clinical symptoms of attention-deficit/hyperactivity disorder: a feasibility study	South Korea	Sun et al. [76]	30	27	Children	Nurow	Digital therapeutic interventions	CAT PSYTOOLKIT CBCL	28	None
Predictors and moderators of treatment outcome in cognitive training for children with ADHD	Norway	van der donk et al. [77]	98	98	Children	Cwmt Pac	Psychological	LDS ODD	25	Working memory
Randomized control study of the effects of executive function training on peer difficulties of children with attention-deficit/hyperactivity disorder c subtype	China	Lan et al. [78]	96	96	Children	Geft Sst	Psychological	SAICA CPT-II WCST Adhd rating scale-iv Raven’s progressive matrices	84	Working memory Cognitive flexibility
Response inhibition and interference control in adult attention deficit hyperactivity disorder	Türkiye	Çelik et al. [79]	85	42	Adults	Neuropsychological	Psychological	Stroop test Bis-11 Ibm spss version 23.0 Sst	1095	Inhibition
Scaffolding the attention-deficit/hyperactivity disorder brain using transcranial direct current and random noise stimulation: a randomized controlled trial	Israel	Berger et al. [80]	19	19	Children	Transcranial electrical stimulation Computerized of training	Digital therapeutic interventions	TDCS DLPFC	365	Working memory
Safety and efficacy of guanfacine extended-release in adults with attention deficit/hyperactivity disorder: an open-label, long-term, phase 3 extension study	Japan	Iwanami et al. [81]	150	150	Adults	Grx	Medication	CAARS CGI-I BRIEF	350	Working memory
Randomized controlled study on the effect of multimodal therapy and drug therapy on children with attention deficit hyperactivity disorder	China	Qian et al. [82]	61	61	Children	Medicine and Psychological treatment	Multimodal Treatment	SNAP STROOP WCST	360	Working Memory Inhibition
Moderating role of individual and familial characteristics in the improvement of organizational skills ADHD Youths’ participation in the TRANSITION project	Canada	Girars-Lapointe et al. [83]	70	70	Children	Medicine and Psychological treatment	Multimodal Treatment	DISC-IV Conners WISC-IV	270	Planning Working Memory Organization
